# Geographic profiling applied to testing models of bumble-bee foraging

**DOI:** 10.1098/rsif.2008.0242

**Published:** 2008-07-29

**Authors:** Nigel E. Raine, D. Kim Rossmo, Steven C. Le Comber

**Affiliations:** 1 Research Centre for Psychology, School of Biological and Chemical Sciences, Queen Mary, University of LondonMile End Road, London E1 4NS, UK; 2 Department of Criminal Justice, Texas State University-San MarcosSan Marcos, TX 78666, USA; 3 School of Biological and Chemical Sciences, Queen Mary, University of LondonMile End Road, London E1 4NS, UK

**Keywords:** animal foraging behaviour, bumble-bee *Bombus terrestris*, criminology applications, jeopardy surface, spatial foraging strategies, theoretical foraging algorithms

## Abstract

Geographic profiling (GP) was originally developed as a statistical tool to help police forces prioritize lists of suspects in investigations of serial crimes. GP uses the location of related crime sites to make inferences about where the offender is most likely to live, and has been extremely successful in criminology. Here, we show how GP is applicable to experimental studies of animal foraging, using the bumble-bee *Bombus terrestris*. GP techniques enable us to simplify complex patterns of spatial data down to a small number of parameters (2–3) for rigorous hypothesis testing. Combining computer model simulations and experimental observation of foraging bumble-bees, we demonstrate that GP can be used to discriminate between foraging patterns resulting from (i) different hypothetical foraging algorithms and (ii) different food item (flower) densities. We also demonstrate that combining experimental and simulated data can be used to elucidate animal foraging strategies: specifically that the foraging patterns of real bumble-bees can be reliably discriminated from three out of nine hypothetical foraging algorithms. We suggest that experimental systems, like foraging bees, could be used to test and refine GP model predictions, and that GP offers a useful technique to analyse spatial animal behaviour data in both the laboratory and field.

## 1. Introduction

Geographic profiling (GP) is a statistical technique originally designed to help police forces to prioritize large lists of suspects typically generated in cases involving serial crime, for instance, murder and rape (Rossmo 2000). The technique uses the location of related crime sites to make inferences about the most likely area in which the offender might live (or visit regularly), and has been extremely successful in this field (Canter 2003; Santtila *et al*. 2003; Gore *et al*. 2005; Kucera 2005; Sarangi & Youngs 2006; Bennell & Corey 2007; Canter & Hammond 2007). The need for such a technique arises because investigations of serial crimes frequently generate too many, rather than too few, suspects. For example, police investigating the Yorkshire Ripper murders amassed a total of 268 000 names and 4.5 million vehicle registration numbers (Doney 1990; Rossmo 2000). Clearly, lack of time and resources preclude detailed investigation of every suspect in such a case, and it is this problem that GP addresses.

The methods underlying GP depend on two concepts: (i) distance decay and (ii) the buffer zone surrounding the anchor point, normally the criminal’s or animal's home (Rossmo 2000; Le Comber *et al*. 2006*a*). The first concept relies on the fact that most crimes occur relatively close to the criminal's home (e.g. 70% of arsons occur within 2 miles of the arsonist's home; Sapp *et al*. 1994) because travelling usually incurs costs in time, effort and/or money. However, the criminal's home is also typically surrounded by an area (the buffer zone) in which offences are relatively rare. This buffer zone arises partly because of increases in detection risk related to reduced anonymity within the criminal's local neighbourhood, and partly because the number of criminal opportunities increases geometrically with distance travelled from home. The size of the buffer zone is therefore specific to an individual. Criminology studies suggesting the absence of a buffer zone are the erroneous attempt to infer individual-level characteristics from an average group characteristic. This is an ecological fallacy and treating distance data in this manner distorts the shape of the distribution (van Koppen & de Keijser 1997). For example, Warren *et al*. (1995) failed to find a buffer zone in their first analysis of aggregated serial rape journey-to-crime (JTC) data. However, when they used normalized individual-level JTC distributions, they observed a distinct buffer zone (Warren *et al*. 1998).

GP uses the opposing effects of distance decay and the buffer zone to calculate the probability of offender residence for each location within the study area, producing a three-dimensional probability surface (called a jeopardy surface). Locations in which it is more likely that the offender might live are indicated by higher points on the jeopardy surface. Overlaying the three-dimensional jeopardy surface onto a search area map produces a geoprofile. Hence, geoprofiles do not provide an exact location for the criminal's home, but they allow the police to prioritize search locations by starting with the highest point on the jeopardy surface. Systematically checking locations in descending order of their height on the geoprofile probability surface describes an optimal search process based on decreasing probability density. Therefore, the better the GP model performs, the shorter the search before the real location of the offender's home is found.

The success of GP in criminology (Canter 2003; Santtila *et al*. 2003; Gore *et al*. 2005; Kucera 2005; Sarangi & Youngs 2006; Bennell & Corey 2007; Canter & Hammond 2007) has recently led to this technique being applied to the foraging behaviour of bats (Le Comber *et al*. 2006*a*) and great white sharks (R. A. Martin *et al*. 2004, unpublished data). In both these studies, GP techniques were retrospectively applied to natural foraging data, originally collected for different studies, to test the efficacy of this technique on animal data. As the technique has now been shown to be useful in such divergent scenarios from those for which it was originally developed, it raises the intriguing possibilities that GP could be a useful general tool for studying animal foraging and also that experimental manipulation of animal foraging might be usefully used to refine GP techniques. Therefore, we set out to apply GP techniques to compare the foraging patterns of bumble-bees (*Bombus terrestris* L.) in a laboratory experiment with simulated patterns produced using a range of computer foraging algorithms.

The behavioural traits of social bees that make them model organisms for foraging studies also make them well suited to tests of GP. It has even been suggested that colonies could maintain a buffer zone immediately around their nest entrance, in which they do not forage, that could act to reduce the chance of predators and parasites locating the nest (Dramstad 1996; Saville *et al*. 1997). The literature on foraging in bees is extensive (e.g. Heinrich 1979; Schmid-Hempel 1985; Dramstad 1996; Raine *et al*. 2006*a*; Raine & Chittka 2008), and various hypothetical foraging strategies have been proposed. These include both nearest neighbour movements and linear searches, both of which have been observed in bees foraging on real (Pyke 1978; Zimmerman 1979) and artificial flowers (Schmid-Hempel 1985; Pyke & Cartar 1992). One aim of this paper is to use GP to compare patterns of foraging in naive *B. terrestris* workers with patterns produced by ‘virtual’ bees using different foraging algorithms (including nearest neighbour movements and linear searches), and to determine which of these hypothetical foraging strategies are compatible with the experimental data.

In this study, we fit model variables to known anchor points (in criminology, usually the criminal's residence or work place, to which they return between crimes, but in animal foraging the location of a nest, roost or den) for both experimental and simulated foraging data. We use these variables as the basis for hypothesis tests to compare patterns of foraging at different densities of food items (flowers), and to compare different foraging algorithms. We go on to compare actual bee foraging with that expected under different models of foraging behaviour. Specifically, we address four questions.

1. Can GP be used to locate the nest entrance using the observed patterns of flower visitation by foraging bees?
2. Can GP be used to discriminate between patterns of foraging arising from differences in the densities of potential food items (flowers), in either (i) real bees or (ii) computer simulations?
3. Can GP be used to discriminate between patterns of foraging arising from different simulated foraging strategies?
4. Can GP be used to compare the actual foraging behaviour of *B. terrestris* with that expected under different hypothetical models of foraging?

## 2. Methods

### 2.1 Experimental study

The bumble-bee (*Bombus terrestris canariensis* Pérez) colony, obtained from a commercial bee breeder (Koppert Biological systems, Berkel en Rodenrijs, The Netherlands), was housed in a wooden nest-box (28×16×11 cm) and fed defrosted pollen (Koppert Biological systems) and artificial nectar (Apiinvert, E. H. Thornes, UK) ad libitum prior to experiments. During the experiment, the nest-box was connected to a wooden flight arena with a colourless transparent Plexiglas lid (100×100×35 cm), via a colourless Plexiglas tube opening in the centre of the arena floor. Bee traffic between the nest and arena was controlled using shutters within this tube. Initially, bees were allowed unrestricted access to the flight arena and presented with a colourless glass feeder (containing ad libitum 40% sucrose solution (w/w)) placed at a random location. Bees landing on the feeder were marked with individually numbered, coloured tags (Opalith tags, Christian Graze KG, Germany), a standard procedure to allow experimenters to recognize individual insects for behavioural observations. The marking procedure involved capturing a bee with forceps, then transferring it to a marking cage (E. H. Thornes, UK) in which it is temporarily immobilized to allow accurate tag application on the dorsal thorax region. The tagged bee was then returned to the nest-box, and appeared to suffer no long-lasting effects of the procedure. We monitored which marked bees returned regularly to the feeder to ensure only motivated foragers were tested. Controlled illumination for all experiments was provided by high-frequency fluorescent lighting (TMS 24F lamps with HF-B 236 TLD (4.3 kHz) ballasts (Philips, The Netherlands) fitted with Activa daylight fluorescent tubes (Osram, Germany)), which simulates natural daylight above the bee flicker fusion frequency.

Subsequently, the foraging behaviour of each motivated forager was observed for a single foraging bout when presented with blue artificial flowers in the arena. Each flower comprised a plastic square (Perspex Blue 727: 24×24 mm) standing on a vertical glass cylinder (diameter 10 mm and height 40 mm). Flowers each contained 10 μl of 40 per cent sucrose solution (w/w), placed in the centre of the square, and were placed at randomly generated coordinates within a 33×33 grid in the arena (grid cell dimensions 30.3×30.3 mm). After entering the arena via the central hole in the floor (coordinates 17, 17), each bee could forage freely and we recorded the coordinates of all the flowers from which it fed, and the order in which it visited them. Bee foraging behaviour was observed for two flower densities (*n*=32 or 64 flowers), testing 12 individuals at each density (*n*=24 bees). Bees were tested individually, using different random flower positions for each individual. As bees were not exposed to colour stimuli associated with food or the spatial locations of artificial flowers before the experiment they can be considered naive with respect to this foraging task (Raine *et al*. 2006*b*; Raine & Chittka 2007*a*). The 24 bees tested each fed on 10–21 flowers (median 17.5; mean 16.9). Between tests, the arena was wiped down with 70 per cent ethanol (v/v) and then with distilled water, and the flowers replaced with fresh ones placed in new random positions to prevent bees using odour cues left by previous foragers to inform their flower choices (Saleh *et al*. 2006).

### 2.2 The model

The model is a modification of the equations given in Rossmo (2000) and Le Comber *et al*. (2006*a*), which uses Euclidean, rather than Manhattan, distances. Manhattan distances are commonly used in criminological applications of GP as movements in urban environments in the United States follow grid-like street patterns. In this study, we use Euclidean distances since there is no *a priori* reason to assume that animals will follow particular routes between locations. As the ratios between the metrics (Manhattan or Euclidean distance) vary within a limited range, the issue will not cause problems, providing of course that the same metric is used throughout a study.

**Figure 1:**
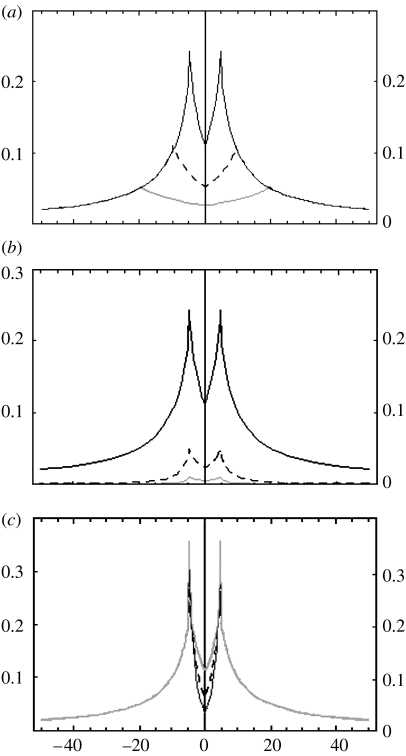
The effects of altering the model variables (*a*) *B*, (*b*) *f* and (*c*) *g* on the shape of the jeopardy surface (shown here as a two dimensional slice through the caldera-shaped function output). The *x*-axis shows the distance (in arbitrary units) from the anchor point, and the *y*-axis shows the height of the jeopardy surface (the probability of offender residence); in each case, there is a single crime site at point *x*=0. Outside the buffer zone radius (*B*), probability of offender residence drops with distance from the crime location. Within the buffer zone radius, probability of offender residence drops with proximity to the crime location. Thus, residence probability is highest at the buffer zone radius. Default values for each panel are *B*=5; *k*=1; *f*=1; *g*=1: (*a*) *B* set to 5 (solid line), 10 (dashed line) and 20 (grey line); (*b*) *f* set to 1 (solid line), 2 (dashed line) and 3 (grey line); (*c*) *g* set to 1 (solid line), 2 (dashed line) and 3 (grey line).

For each point (coordinates *i*, *j*) within the study area, the score function *p* was calculated as follows:

(2.1) $$p_{ij}=k\sum_{n=1}^{C}\left[\frac{\phi }{{\left(\sqrt{{\left(x_{i}-x_{n}\right)}^{2}+{\left(y_{j}-y_{n}\right)}^{2}}\right)}^{f}}+\frac{\left(1-\phi )(B^{g-f}\right)}{{\left(2B-\sqrt{{\left(x_{i}-x_{n}\right)}^{2}+{\left(y_{j}-y_{n}\right)}^{2}}\right)}^{g}}\right],$$

where

(2.2) $$\sqrt{{\left(x_{i}-x_{n}\right)}^{2}+{\left(y_{j}-y_{n}\right)}^{2}}>B⊃\phi =1$$

and

(2.3) $$\sqrt{{\left(x_{i}-x_{n}\right)}^{2}+{\left(y_{j}-y_{n}\right)}^{2}}\leq B⊃\phi =0,$$

such that *ϕ* functions as a weighting factor that is set to 0 for sites within the buffer zone, and 1 for sites outside the buffer zone. *k* is an empirically determined scaling constant (which affects the vertical scale of the jeopardy surface but not its shape); *B* is the buffer zone radius (given as number of grid square units: 1 unit=30.3 mm as in the experimental study); *C* is the number of foraging sites; *f* and *g* are empirically determined exponents that dictate the form of the curve as it approaches, and then moves away from, the radius of the buffer zone (figure 1); (*x*_*i*_, *y*_*j*_) are the coordinates of point (*i*, *j*); and (*x*_*n*_, *y*_*n*_) are the coordinates of the *n*th foraging site. Thus, *p*_*ij*_ describes the likelihood that the anchor point occurs at point (*i*, *j*), given the foraging site locations.

The equation describes a two-part curve. Outside the buffer zone radius, probability of offender residence drops with distance from the crime location. Within the buffer zone radius, probability of offender residence drops with proximity to the crime location. Thus, probability is maximal at the buffer zone radius: because the function extends in all directions, in three dimensions, the function output resembles a volcano caldera (figure 1).

#### 2.2.1 Model fitting

We set *k*=1, and fitted values of *B*, *f* and *g* for each replicate (bee tested) within the experimental study (using flower densities=32 and 64), and for each simulated dataset (see below). The buffer zone radius, *B*, was calculated as the mode of the straight line distances between each selected flower and the arena entrance for each bee (Rossmo 2000): distances were rounded to nearest integer to facilitate this calculation. A mean value of *B* was then calculated for each dataset by averaging across the 12 bees in each dataset. The variables *f* and *g* were empirically determined, with the constraint *f*=*g* (this is common in criminal cases, where *f* and *g* are set to 1.2; Rossmo 2000). For each model replicate (bee), we tested *f* and *g* values between 0.1 and 2 in increments of 0.1, and calculated the model's hit score percentage (see below) in each case (note that since *f*=*g*, only *f* values are reported in the results). Model performance is measured by the model's hit score percentage: given by the ratio of the total number of points (grid squares) with scores higher than that of anchor point (arena entrance), plus half the number of points with equal scores, to the total number of points included within the search area. The assumption is that, on average, half the points of equal height would have to be searched before the anchor point was found. When the jeopardy (probability) surface is flat (i.e. there is no reason to prioritize the search to begin at any location, and the search is essentially uniform), the mean hit score is 50 per cent. This means that an average search would be expected to locate the anchor point after covering half the total search area. Mean hit scores can range from 0 (optimal performance) to 50, the value expected by chance, or above (worse than random). In all cases, lower hit score values indicate a more efficient search process. Fitted *f* and *g* values used were those that described the most efficient search procedure (i.e. those with the lowest hit score); where two or more *f* and *g* values were equally efficient, the mean value was used. The hit score produced by model fitting is the learning hit score (table 1). All foraging algorithms, GP models and statistical analyses used in this study were carried out in Mathematica v. 5.0 (Wolfram Research Inc., IL, USA) running on a Dual 2 GHz PowerPC G5 Macintosh desktop computer.

#### 2.2.2 Model validation

The model fitting process yielded values of *B*, *f* and *g* for each replicate (bee), which we used to derive inputs to validate the model. For each replicate (bee), the model was tested using mean *B*, *f* and *g* values derived from the remaining replicates (bees) for that foraging algorithm (or the experimental data) and flower density. This ensured that the testing and learning sets were independent. For example, when using 12 test bees, we used the *B* and *f* (=*g*) values for bees 1 and 3–12 in order to validate the model fits to bee 2. Hit scores were recalculated to produce a test hit score (table 1). Thus, the learning hit score relates to the process when the model variables are fitted to the data (for one bee), while (independent) test hit scores are the hit score percentages when the model was validated using mean *B*, *f* and *g* values derived from the remaining (*n*=11) bees tested.

#### 2.2.3 Simulation 1

**Figure 2:**
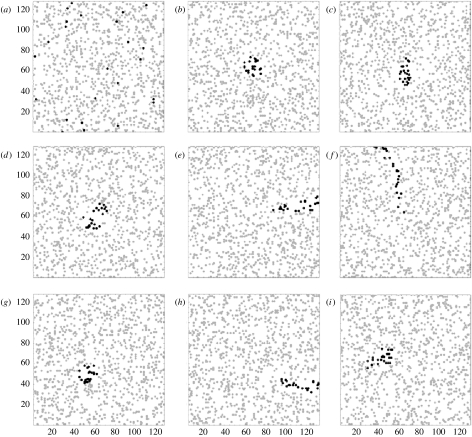
Example patterns of flower choices made by a virtual bee following nine different theoretical foraging algorithms in simulation 2. In each case, the virtual bee forages in a grid containing 1024 flowers (grey circles), of which it selects 24 (black circles). The foraging algorithms shown are: (*a*) random (R); (*b*) nearest neighbours (NN); (*c*) shortest steps (SS); (*d*) spiral (Sp); (*e*) linear 1 (Li_1_); (*f*) linear 2 (Li_2_); (*g*) nearest neighbours displaced (NN_d_); (*h*) shortest steps displaced (SS_d_); and (*i*) spiral displaced (Sp_d_). For further details of the foraging algorithms, see §2.

We applied eight different hypothetical foraging algorithms to the same foraging tasks presented to the real bumble-bees (i.e. simulation conditions matched those of the experimental study). Flower distributions were simulated by random allocation to cells within a 33×33 grid in exactly the same way as in the experimental study (N.B. flowers could not be positioned at coordinates 17, 17 as this is the arena entrance hole). Similarly, the replicate number matched those in the experimental study: for each of the eight foraging algorithms, we ran 12 replicates (virtual ‘bees’) at each food density (*n*=32 or 64 flowers), and each replicate (bee) selected 16 flowers from the array. Flower distributions were re-randomized for each replicate. Foraging algorithms were defined as follows (figure 2).

- *Random* (*R*). Bees choose each flower entirely at random from the array.
- *Nearest neighbours* (*NN*). Bees choose the 16 flowers nearest to the arena entrance (coordinates 17, 17).
- *Shortest steps* (*SS*). Bees first choose the flower nearest the arena entrance, then choose the nearest flower not previously selected at each step.
- *Spiral* (*Sp*). Flowers are allocated to constant width (eight cells) concentric bands surrounding the arena entrance, and ordered according to their angle from the arena entrance. Starting with the flower nearest the arena entrance, bees search the innermost band consecutively from 0 to 360° before moving to the nearest flower in the next concentric band and continuing to search in a clockwise direction.
- *Linear 1* (*Li_1_*). Flowers selected in a randomly chosen arena sector, such that the selected flowers describe a ‘wedge’-shaped area.
- *Nearest neighbours displaced* (*NN_d_*). As NN, but the bee chooses the first flower at random.
- *Shortest steps displaced* (*SS_d_*). As SS, but the bee chooses the first flower at random.
- *Spiral displaced* (*Sp_d_*). As Sp, but the bee chooses the first flower at random.

For each set of replicates (bees, or virtual bees) at each combination of flower densities and foraging algorithms, the GP model was fitted and validated (producing learning and test hit scores, respectively) as above. Fitted values were compared using *t*-tests, Bonferroni corrected for multiple comparisons.

#### 2.2.4 Simulation 2

This simulation used a similar design as the first, but with a larger foraging arena (129×129 cells) and higher flower densities (1024 and 2048, respectively); each virtual bee selected 24 flowers per foraging bout. Arena dimensions for this simulation are arbitrary, and were simply chosen to produce a larger arena size than used in simulation 1 (and the experimental study). We used the same foraging algorithms as simulation 1, with one addition.

- *Linear 2* (*Li*_2_). The bee selects the flower nearest the arena entrance, and its angle from the arena entrance is calculated. The next flower chosen is that nearest the previous food item in the same direction (±30°) as the first step. This foraging algorithm was not used in simulation 1, since the smaller grid size and lower flower densities meant that the edge of the arena was generally reached before the algorithm had identified the required 16 flowers (figure 2).

For each set of replicates (virtual bees), at each combination of densities and foraging algorithms, the model was fitted and validated as described above. Fitted values were compared using *t*-tests, Bonferroni corrected for multiple comparisons.

## 3. Results

Fitted values for the buffer zone radius (*B*) and the curve exponents (*f* and *g*) for the experimental data, and for each simulation, are shown in table 1, along with the learning and test hit scores, which provide measures of performance for the model fitting and validation processes, respectively. For experimental data, and for all hypothetical foraging algorithms in simulations 1 and 2, mean test hit scores were significantly lower than the value that defines a random search (test hit score=50; i.e. in a random search of all possible locations in the target area, the actual anchor point is sometimes found first, sometimes last and on average after 50% of the list has been searched) at each density, indicating a very high degree of model validity (Pr<0.001 for all *t*-tests, after Bonferroni correction for multiple comparisons). This indicates that in all cases prioritizing search locations using the GP model means the arena entrance (anchor point) is found much more efficiently than by a random search process (question 1). Test hit scores indicate the model's efficiency in finding the arena entrance relative to random search. So a test hit score of 25 indicates that the model finds the arena entrance in half the time, and a test hit score of 0.1 indicates that the model finds the entrance 500 times faster than a random search (hit score=50). This confirms that GP can be used to speed up the process of locating the arena entrance from the observed patterns of flower visitation by foraging bees.

### 3.1 Experimental study

**Figure 3:**
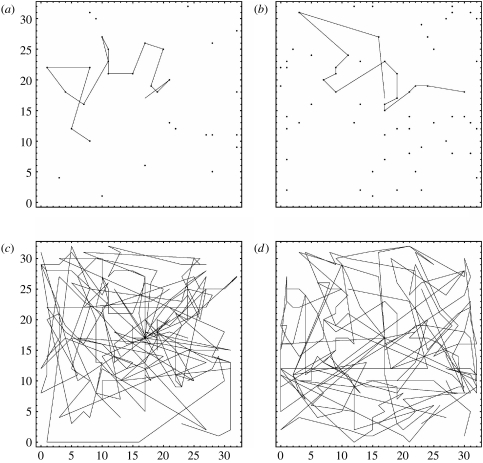
Foraging choices made by real bumble-bees in the experimental study. Data presented show the locations of flowers visited by two example individual bees when foraging at flower densities of (*a*) 32 and (*b*) 64 flowers. The positions of all flowers are indicated by black dots, and the choice sequence of each bee is indicated by the black line (although for simplicity this is indicated by a straight line between consecutive flower choices, this does not indicate the real path flown between flowers). The flower choice sequences are shown overlaid for all 12 bees tested at each flower density, i.e. (*c*) 32 and (*d*) 64 flowers (here the positions of unvisited flowers are not shown for clarity). Each bee entered from the centre of the arena (coordinates 17, 17) and selected 16 of the flowers available.

Observing bees foraging on artificial flowers at different densities (*n*=32 and 64 flowers) in a flight arena, we can see clear differences in their foraging paths (figure 3). Can GP also reliably discriminate between the flower visitation patterns of bees foraging at these same two flower densities (question 2(i))? There were no differences in the fitted values of either *B* or *f* between the two flower densities in the experimental study, when using the entrance to the foraging arena (coordinates 17, 17) as the anchor point (table 1; *B*: *t*=0.209, d.f.=22, Pr=0.418; *f*: *t*=0.1566, d.f.=22, Pr=0.132). Bees chose their first flower in the arena in an apparently haphazard fashion, often flying over several other flowers closer to the arena entrance on the way. The rank distance of the first flower selected (ranking the closest flower to the arena entrance as 1) was 3.5 or 24.5 (median), 7.2 or 28.3 (mean) or between 1–24 and 3–63 (range) at densities of 32 and 64 flowers, respectively (*n*=12 bees at each flower density). We therefore reanalysed these foraging data using the spatial mean of flower locations visited as the anchor point to remove the effect of this initial haphazard movement from the arena entrance. Interestingly, when we considered the spatial mean of the flower locations visited by each bee as the anchor point, the buffer zone radius (*B*) was significantly lower for the high-density treatment (mean ±s.d.: low density, *n*=32 flowers: 9.6±1.71; high density, *n*=64 flowers: 7.1±2.02; *t*=3.24, d.f.=22, Pr=0.002), but fitted *f* values did not differ between treatments (low density, *n*=32 flowers: 1.23±0.187; high density, *n*=64 flowers: 1.18±0.058; *t*=0.88, d.f.=22, Pr=n.s.). This suggests that the GP model fitted to real bee foraging data is sensitive to differences in flower density, and that the buffer zone radius (*B*) can be used to discriminate between them. These differences become apparent when the effect of each bee's first flight, between arena entrance and their first chosen flower, is removed. The distances flown on these first flights were highly variable among bees tested at the same flower density, and did not differ between treatments (mean±s.d. grid square units: low density, *n*=12: 13.38±4.910; high density, *n*=12: 11.63±5.198; *t*=0.85, d.f.=22, Pr=0.41).

### 3.2 Simulation 1

Can GP also reliably discriminate between hypothetical patterns of foraging arising from differences in the density of potential food items in simulation 1 (question 2(ii))? Of the eight hypothetical foraging algorithms run in simulation 1, three (NN, SS and Sp) revealed significant differences in *B* between flower densities (Pr<0.05 in all cases). However, none of these models showed significant differences in *f* values fitted at either low or high flower density.

**Figure 4:**
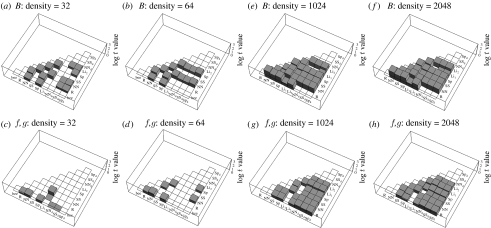
Half matrices showing log *t* statistics for comparisons of *B* (the buffer zone radius: *a*,*b*,*e*,*f*) and *f* (*c*,*d*,*g*,*h*) among different foraging algorithms in simulation 1 plus experimental data (flower densities 32 and 64 (*a*–*d*)) and simulation 2 (flower densities 1024 and 2048 (*e*–*h*)). Only the results of *t*-tests revealing significant differences following Bonferroni correction for multiple comparisons are shown (as grey columns). The height of each column indicates the log *t* value for each of these pairwise comparisons. Keys are the same as in figure 2, plus bee=experimental data. N.B. since *f*=*g*, only *f* values are reported here.

Can GP also be used to discriminate between patterns of foraging arising from different simulated foraging strategies (question 3)? Results of the comparisons among the eight hypothetical foraging algorithms and the foraging data from real bees are shown for fitted values of *B* and *f* in figure 4*a–d*. At the lower flower density (*n*=32), 14 of the 28 pairwise comparisons among the eight computer-generated foraging algorithms could be discriminated from one another using fitted values of either *B* (figure 4*a*) or *f* (figure 4*c*). *B* was more informative than *f*, successfully differentiating between 13 algorithm pairs, compared with two pairs for *f* (N.B. counts exclude comparisons with the experimental bee data; see below). At the higher flower density (*n*=64), 12 of 28 pairwise comparisons could be discriminated using fitted values of *B* (figure 4*b*) or *f* (figure 4*d*). Once again, *B* was more informative than *f*, differentiating between 12 versus 4 algorithm pairs, respectively. None of the three ‘displaced’ foraging algorithms (NN_d_, SS_d_ and Sp_d_) could be discriminated from one another at either flower density.

### 3.3 Simulation 2

Can GP also reliably discriminate between hypothetical foraging patterns arising from differences in potential food item density in simulation 2 (question 2(ii))? Of the nine foraging algorithms in simulation 2, four (NN, SS, Sp and Li_2_) revealed significant differences in *B* between flower densities (Pr<0.0001 in all cases after correction for multiple comparisons). Only in Sp was a significant difference detected in *f* values fitted at low and high flower density (Pr<0.0001 after correction for multiple comparisons).

Can GP be used to discriminate between foraging patterns produced by different simulated strategies (question 3)? Results of the comparisons among the nine hypothetical foraging algorithms are shown for fitted values of *B* and *f* in figure 4*e–h*. At both the low (*n*=1024) and high flower densities (*n*=2048), all of the foraging algorithms can be discriminated on the basis of fitted values of *B* and *f*, except the three displaced foraging algorithms (NN_d_, SS_d_ and Sp_d_). At low flower density, 28 of 36 pairwise comparisons could be successfully differentiated using fitted values of *B*, the only exceptions being comparisons among the three displaced foraging algorithms, between R and the three displaced foraging algorithms, and between Li_1_ and NN_d_ and Li_1_ and SS_d_ (figure 4*e*). Likewise, *f* values differentiated between 27 of 36 pairwise comparisons, the exceptions being comparisons among the three displaced foraging algorithms and between Li_1_ and both R and NN, and between Li_2_ and both SS and Sp (figure 4*g*). At high flower density, 29 of 36 pairwise comparisons could be successfully differentiated using fitted values of *B*, the only exceptions being comparisons among the three displaced foraging algorithms, between R and the three displaced foraging algorithms, and between Li_1_ and NN_d_ (figure 4*f*). Fitted *f* values differentiated among 29 of 36 pairwise comparisons, the exceptions being comparisons among the three displaced foraging algorithms, between Li_1_ and both R and NN, and between Li_2_ and SS (figure 4*h*). In contrast to simulation 1, here *B* and *f* were almost equally informative when attempting to discriminate between foraging algorithms.

### 3.4 Experimental study versus simulation 1

Can GP be used to compare the actual foraging behaviour of real bees with that expected under different hypothetical models of foraging (question 4)? Results of the comparisons among the eight hypothetical foraging algorithms and the foraging data from real bees are shown for fitted values of *B* and *f* in figure 4*a–d*. The foraging patterns of the real bees can be discriminated from the hypothetical foraging algorithms R, NN and Sp at both low and high densities, and from NN_d_ and SS_d_ at low density, on the basis of either *B*, *f* or both.

## 4. Discussion

Our results indicate that GP can be used to locate the entrance to the flight arena (i.e. functionally the colony nest entrance) from foraging data (question 1) and to discriminate between foraging patterns arising from different densities of potential food items in both experimental (question 2(i)) and simulated (question 2(ii)) data. We also show that GP can be used to differentiate between hypothetical foraging strategies (question 3) and to compare actual foraging behaviour with that expected using different hypothetical foraging algorithms (question 4).

The GP approach offers several advantages over simpler measures of spatial central tendency for predicting the entrance to the flight arena or location of a criminal's home. These measures (e.g. spatial mean) can only produce a single location. However, the GP model assigns a likelihood value for every location within the search area, thereby generating a probability surface (summarized as the geoprofile). Direct comparisons of the predictive capability of the spatial mean and the Criminal Geographic Targeting algorithm used in GP indicate that the latter approach generates a search strategy approximately three times as efficient as the former (Rossmo & Velarde 2008). In addition, the GP model also copes better than the spatial mean in instances in which more than one anchor point exists (e.g. in criminology, the criminal's home and the place where they buy drugs; in biology, multiple bat roosts, aardvark dens or chimpanzee nests).

In this study, GP successfully discriminated among many different hypothetical foraging algorithms and also real bee foraging data. The models were robust, with the results of two simulations being broadly in agreement, despite large differences in the number of flowers used in each scenario. In each case, fitting the GP model usually successfully discriminated among different foraging algorithms. The principal exceptions were the three foraging rules in which the initial choice of flower was displaced from the arena entrance (NN_d_, SS_d_ and Sp_d_), meaning that the random component of the initial step (the flight from the arena entrance to the first flower) obscured differences in subsequent steps (flights between flowers). In general, the higher the flower density, the better the model performed at differentiating between pairs of foraging algorithms. This is not surprising, since at low flower densities, when foragers have fewer choices, different foraging algorithms will tend to select a similar set of flowers. In fact, at the two highest flower densities (1024 and 2048), the model successfully differentiated between all foraging algorithms except the comparisons between the displaced strategies (NN_d_, SS_d_ and Sp_d_). Similarly, in both simulations, NN, SS and Sp showed differences in *B* with flower density, indicating that bees forage closer to the anchor point at the higher flower density. The congruence between the results in both simulations suggests that the model is robust with respect to such changes.

What does GP tell us about bee foraging strategies? First, it is encouraging that the model provides a useful description of bee foraging behaviour, despite obvious behavioural differences between bees and criminals. For example, the GP model was originally developed to describe cases in which criminals return to their residence between crimes, but foraging bees often visit hundreds of flowers during each foraging bout before returning to their nest (Heinrich 1979; Raine *et al*. 2006*a*). Thus, although the first flower is selected in a similar way as a crime site, i.e. with respect to the animal's/criminal's home, the choice of each subsequent flower visit is influenced more strongly by the locations of the previous flowers visited than the nest entrance location. One of the fundamental assumptions of GP developed for criminology is that ‘the target backcloth is not patchy’ (Rossmo 2005). While the spatial location of flowers (artificial and virtual) in these experiments was non-patchy, in that flower position was generated randomly (in contrast to the work of one of the authors on foraging in mole-rats; Le Comber *et al*. 2006*b*), the patchiness of real flowers at the landscape scale over which bees forage is variable and habitat dependent. Data obtained from the waggle dances of honeybee (*Apis mellifera* L.) foragers indicate that the distribution of floral resources is patchier in tropical forests (Dornhaus & Chittka 2004) compared with temperate habitats (Visscher & Seeley 1982; Waddington *et al*. 1994; Beekman & Ratnieks 2000), although temperate habitats also differ in patchiness depending on the degree of disturbance. The effect of variation in the degree of patchiness of potential targets (here flowers) on the applicability of GP is an obvious extension of the study described here, since one of the most exciting elements of applying GP to biological data is that it allows experimental manipulation of conditions in ways that are not possible in criminology.

The small scale of our flight arena in comparison with the flight range of foragers from field bumble-bee colonies (Walther-Hellwig & Frankl 2000; Westphal *et al*. 2006; Wolf & Moritz 2008; Osborne *et al*. 2008) means our results will be most relevant to foraging strategies on small spatial scales, such as those observed within flower patches (Burns & Thomson 2006; Chittka & Raine 2006). Despite these potential limitations, comparing patterns of foraging in the experimental study with patterns produced by virtual bees using different foraging algorithms did allow us to determine which of these hypothetical foraging strategies were compatible with the experimental data (however, it did not allow us to determine which of the remaining hypothetical algorithms are most consistent with the foraging patterns of real bees). Patterns of foraging in the experimental study could be discriminated from the random, nearest neighbour and spiral algorithms at both low and high flower densities, and from two of the displaced foraging algorithms (NN_d_ and SS_d_) at low density but not from the remaining algorithms. These results agree with the literature on bee foraging strategies at small (within flower patch) spatial scales. Bees foraging in monospecific flower patches have been observed using strategies corresponding to the algorithms we defined as ‘shortest steps’, ‘linear 1’ and ‘linear 2’, which could not be discriminated from the foraging behaviour of real bees in this study. For example, bees have been reported making sequences of flights among nearby flowers (Waddington & Heinrich 1981; Zimmerman 1982; Soltz 1986; Kipp *et al*. 1989), and/or maintaining relatively constant flight direction when making sequences of flower visits (Waddington 1980; Schmid-Hempel 1985; Pyke & Cartar 1992). Such simple movement rules could easily increase foraging efficiency by minimizing the travel time between flowers and also the chances of revisiting a flower already recently emptied by the same individual bee (Levin *et al*. 1971; Pyke 1978; Zimmerman 1979; Bell 1991).

Interestingly, our results from foraging bees are not compatible with the spiral or nearest neighbour algorithm. This is surprising, since each of these would mean that bees would tend to choose flowers relatively close to the arena entrance. The fact that bees do not appear to use these strategies might provide support for the idea that they maintain a buffer zone around the nest entrance (Dramstad 1996; Saville *et al*. 1997), but we found no difference in the buffer zone radius (*B*) between flower densities (*n*=32 versus 64) when we used the arena entrance as the anchor point. However, *B* was significantly lower for the high flower density (*n*=64) when the spatial mean of the flower locations chosen by the bee was taken as the anchor point. This suggests that differences in foraging patterns between these flower densities are masked by the initial longer flight between the arena entrance and the first visited flower when considering the arena entrance (rather than the spatial mean of flower locations) to be the anchor point. The existence of a buffer zone, immediately surrounding the nest entrance, is also inconsistent with data from other studies; for example, *B. terrestris* workers did not appear to avoid flowers closer to the arena entrance in laboratory experiments at a similar spatial scale (Raine *et al*. 2006*b*; Saleh *et al*. 2006), and *B. terrestris* workers have also been observed visiting flower patches within 3 m of their nest entrance under natural conditions (T. C. Ings 2006, personal communication). In addition, even if workers were not foraging close to the nest entrance (i.e. maintaining a buffer zone), it seems likely that predators and parasites would be alerted to general nest location by regular flow of forager traffic entering and leaving and would then home in more precisely using colony scent.

Our results support conclusions drawn from criminological applications of GP in that altering the buffer zone radius (*B*) has significantly more influence on the model than changing the exponents *f* and *g* (Rossmo 2000). Although *B* was more informative than *f* (and *g*) in differentiating between potential foraging strategies when either was considered in isolation (particularly at the smaller of the two spatial scales used; figure 4), using both factors in combination produced the best results in our study. The residual discriminant power of *f* is highlighted when comparing different patterns of foraging which have the same value of *B* and which look quite different, such as when foraging is not radially symmetrical around the nest entrance. For example, the GP model applied to a study of bat foraging in the field performed well when the anchor point (bat roost) was positioned well away from the centre of the study area (Le Comber *et al*. 2006*a*), which effectively skewed the apparent distribution of potential targets (foraging sites).

Our experimental design imposed potential constraints on the possible range of foraging strategies that bees could use. In comparison with their real foraging environment, the small arena size constrains the bees' use of linear-like strategies. Bees attempting to visit sequences of flowers while travelling in a more or less constant direction across the arena (patch) would quickly arrive at the wall—exactly as we found when running the linear 2 algorithm under analogous conditions. The relatively low number of flowers presented to each bee might also restrict their potential use of foraging strategies, particularly the low flower density (*n*=32), from which workers selected between 10 and 21 depending on their size. In the wild, a forager's choices would be considerably wider both in terms of the number of flowers and species on offer (Chittka *et al*. 1997; Raine & Chittka 2007*b*).

However, in spite of the overall simplicity of our experiment, i.e. presenting bees with a single uniformly rewarding flower species, and the limitations of spatial scale, GP allowed us to detect clear differences in foraging behaviour exhibited by bees offered two different flower densities, and between real data and several foraging algorithms. This suggests that future studies could usefully be extended to examine such differences at larger, more ecologically realistic, spatial scales. In this laboratory study, GP appears to work effectively for foraging data when the values of *B* and *f* (=*g*) are invariant. But how applicable are such techniques in more heterogeneous and complex environments such as those in which wild animals forage? The success of applying GP in criminology suggests that the technique is robust to heterogeneous environments, even when the subjects (serial criminals) are actively trying to avoid capture (Rossmo 2000). Radiotelemetry data collected from two bat (pipistrelle) species foraging in the wild also suggest that, despite the undoubted heterogeneity of the natural environment, foraging patterns are sufficiently consistent within species to allow differences between species to be reliably detected using GP (Le Comber *et al*. 2006*a*). The natural level of variation in the spatial distribution of flowers visited by bees is likely to be similar to that shown by the insect prey of these bat species, suggesting that GP is likely to be similarly applicable to study the foraging patterns of bees in their natural habitat. Furthermore, the patterns of great white shark attacks on seal colonies also seem to be well described by GP (R. A. Martin *et al*. 2004, unpublished data). Overall, we feel that these diverse biological examples provide support for the idea that this technique is generally applicable in ecologically realistic scenarios. However, given the small number of studies applying the technique of GP to spatial patterns of animal foraging, it seems clear more work needs to be done in an animal context.

In future, GP could be applied to help locate bee nests, or areas of potential nesting habitat in fragmented landscapes, from the spatial distribution of observed foraging sites alone (as opposed to other interesting but more complex approaches, e.g. Suzuki *et al*. 2007). Locating nest sites of wild bees frequently proves extremely difficult, so any technique which could improve search success would be very valuable, particularly for the increasing number of rare or endangered bumble-bee species. In criminology, the GP model frequently provides very efficient searches when provided with limited numbers of crime sites (Rossmo 2000 suggests a minimum of five). Tracking down nests of rare bumble-bees is a similar proposition, as researchers typically observe only very few foragers within the colony flight range, and nest membership could be identified using non-lethal DNA sampling techniques (Holehouse *et al*. 2003; Châline *et al*. 2004). In addition, GP could be used to compare the foraging strategies of bumble-bee colonies with known nest sites, such as when experimental colonies containing individually marked bees are placed in the field in different habitat types (Chittka *et al*. 2004; Ings *et al*. 2005; Raine & Chittka 2005). This approach could provide useful information about how changes in land usage and habitat fragmentation, both cited as key factors in long-term bumble-bee declines (Williams 1982, 1986; Osborne & Corbet 1994; Carvell *et al*. 2006), affect the patterns of bee foraging on a landscape scale. It would be interesting to use this approach to compare the foraging patterns shown by a given bee species across a range of habitat types/levels of habitat fragmentation (Osborne *et al*. 1999; Westphal *et al*. 2006), and also to compare the foraging patterns across bumble-bee species in similar habitats.

The application of GP to biological data is an exciting and interesting development, and one that is likely to be applicable to a wide range of disparate areas. Given the success of the GP method applied to serial criminals who have shifted their anchor points, e.g. the Yorkshire Ripper, who moved house during the investigation, this approach may potentially be useful for investigating animals which have multiple anchor points: for example, chimpanzee and gorilla groups that regularly shift their home base to follow seasonal food availability in tropical forests. Another possible extension would be to use maximum-likelihood methods to estimate values for *B*, *f* and *g* simultaneously. Possible future uses of GP are not confined to tests of foraging behaviour. Currently, the authors are engaged in studies using GP models to study the distribution patterns of illegal snares in Zimbabwe, and also the origin and spread of invasive species and disease. This technique may also have potential applications for assisting with estimating local species abundance and biodiversity in ecological communities.

In conclusion, we note that experimental systems such as that described here can be used to test the assumptions and predictions of GP in a way that presents obvious logistical and ethical difficulties in criminology. We suggest that, given the considerable success of GP in criminology, where sample sizes are often extremely small and criminals may actively seek to mislead police, it will be surprising if the technique does not prove similarly useful in studying spatial patterns in biology.

## Figures and Tables

**Table 1:** Fitted values for each model variable (*B*, *f* and *g*) along with learning and test hit scores for experimental bee foraging data and for each simulation (1 and 2). (Learning hit scores are the hit score percentages when the model variables are fitted to the data; test hit scores are the hit score percentages when the model was validated using mean *B*, *f* and *g* values derived from the remaining replicates (bees tested). Therefore, learning and test hit scores provide independent measures of performance for the model fitting and validation processes, respectively. Mean hit scores can range from 0 (optimal performance) to 50, the value expected by chance. Values of the learning or test hit score may be compared with the expected value for a random search (50), with lower values representing a more efficient search process in all cases. Model variables *f* and *g* are dimensionless curve exponents (N.B.: *f*=*g*); the buffer zone radius (*B*) is given as number of grid square units (where one unit is 30.3 mm). All values presented are means±1 s.e.)

model	*buffer zone radius* (*B*)		*curve exponents* (*f*,*g*)		learning hit score (mean±1 s.e.)		test hit score (mean±1 s.e.)	
				
experimental study	*n*=32	*n*=64	*n*=32	*n*=64	*n*=32	*n*=64	*n*=32	*n*=64
bees	11.6±0.48	11.4±1.02	1.2±0.04	1.4±0.12	11.5±3.52	6.6±1.75	11.7±10.45	29.9±13.13
simulation 1	*n*=32	*n*=64	*n*=32	*n*=64	*n*=32	*n*=64	*n*=32	*n*=64
R	13.1±0.37	12.1±0.32	0.1±0.01	0.4±0.21	6.0±1.74	10.4±2.52	5.5±6.85	9.6±9.53
NN	8.8±0.27	6.6±0.27	0.1±0.00	0.1±0.00	4.2±1.07	1.8±0.43	4.3±3.86	2.2±2.06
SS	12.3±0.28	10.4±0.57	0.7±0.25	1.0±0.26	17.8±3.49	18.8±3.79	19.4±12.23	23.8±13.65
Sp	8.9±0.28	6.3±0.20	0.4±0.15	0.2±0.05	5.5±1.34	3.8±0.87	6.8±5.37	4.0±3.69
Li_1_	13.2±0.34	11.9±0.49	1.0±0.26	0.8±0.25	21.8±3.01	23.6±4.28	22.6±12.16	29.1±13.50
NN_d_	11.1±0.65	11.0±0.81	0.5±0.21	0.9±0.26	15.2±2.33	18.1±4.62	18.0±10.39	24.6±14.05
SS_d_	10.8±0.54	11.5±0.90	0.4±0.12	0.7±0.23	11.3±3.72	13.5±2.82	15.7±11.76	24.9±17.46
Sp_d_	11.1±0.40	12.3±0.48	0.6±0.22	1.3±0.24	10.6±2.13	16.1±2.74	14.0±8.27	19.0±11.89
simulation 2	*n*=1024	*n*=2048	*n*=1024	*n*=2048	*n*=1024	*n*=2048	*n*=1024	*n*=2048
R	48.2±0.39	49.5±0.35	0.2±0.04	0.2±0.03	7.1±0.65	7.1±0.59	7.7±6.81	7.4±0.63
NN	7.5±0.09	5.2±0.07	0.2±0.04	0.1±0.03	0.1±0.01	0.1±0.01	0.1±0.11	0.1±0.01
SS	15.5±0.41	11.0±0.31	0.7±0.09	0.8±0.09	5.0±0.40	2.6±0.22	5.4±2.95	2.6±0.15
Sp	9.0±0.15	6.6±0.05	0.8±0.08	0.4±0.06	0.4±0.05	0.2±0.01	0.4±0.42	0.2±0.01
Li_1_	44.2±0.30	44.6±0.28	0.1±0.02	0.3±0.00	22.9±0.24	21.3±0.23	23.4±2.60	21.8±0.26
Li_2_	24.6±0.63	17.6±0.39	0.8±0.09	0.8±0.09	14.9±0.83	7.6±0.42	15.7±6.10	7.8±0.29
NN_d_	48.8±1.51	48.8±1.81	1.5±0.08	1.6±0.07	8.1±0.49	6.9±0.39	28.0±16.69	33.2±1.86
SS_d_	48.8±1.68	50.4±1.68	1.4±0.08	1.5±0.08	7.4±0.51	7.6±0.49	28.1±18.08	30.5±1.96
Sp_d_	51.1±1.66	52.0±1.66	1.6±0.08	1.5±0.08	7.9±0.49	7.7±0.46	31.1±18.49	31.6±1.83
